# Monoclonal anti-amyloid antibody treatment: the epidemiological profile of the target patients in Austria and the status of treatment-eligible patients registered at an outpatient memory clinic

**DOI:** 10.1007/s00508-024-02465-8

**Published:** 2024-10-23

**Authors:** Seungjune Lee, Elisabeth Stögmann

**Affiliations:** Department of Neurology, Medical University of Vienna, Waehringer Gürtel 18–20, 1090 Vienna, Austria

**Keywords:** Alzheimer, Dementia, Monoclonal antibody, Epidemiology, Screening

## Abstract

The development of monoclonal anti-amyloid antibodies, a disease-modifying treatment for Alzheimer’s disease (AD), has raised the necessity to identify the epidemiological profile of the possible target patients who would benefit from such therapy. These are patients in the early stages of AD with biomarker-confirmed brain amyloid positivity. In this study, the epidemiological profile of possible target patients in Austria and Vienna was estimated. The number of patients in the stage of amyloid-beta (Aβ)-positive prodromal AD in Austria and Vienna are 193,500 and 34,700 patients, respectively. The expected patient demand for the upcoming therapy in Austria and Vienna are 61,200 and 11,100 patients, respectively.

In the memory clinic of the Vienna General Hospital, the number of treatment-eligible patients for an upcoming anti-amyloid antibody was on average 52.8 patients per year, which is about 10% of the total number of patients visiting the memory clinic every year. Several challenges to provide therapy to the general population include expanding the MCI screening in primary care and increasing the capacity of the healthcare system for biomarker testing, infusion delivery, and ARIA management. The study primarily addresses the status quo of identifying patients on memory clinics through cognitive screening and biomarker testing.

## Introduction

Promising results from recent clinical trials have raised hopes for a breakthrough in disease-modifying treatment for Alzheimer’s disease (AD) in the near future [1, 2]. Currently, there is a rising demand in the healthcare community for the epidemiological data of the population that would benefit from such treatment, including the prevalence of mild cognitive impairment (MCI) patients with positive amyloid-beta (Aβ) biomarkers, the expected number of patients indicated for treatment, and the number of such patients registered in the memory clinics.

## Material and methods

The number of patients in the stage of Aβ-positive prodromal AD in Austria and in Vienna was estimated using epidemiological data from published meta-analyses [3–5]. The number of individuals with MCI was estimated by multiplying the population size by the reported prevalence of MCI in each age group [6]. Additionally, the estimates of MCI patients who are Aβ-positive were determined by applying the Aβ-positivity rate among MCI patients [7]. The expected patient demand for an upcoming AD treatment, e.g., monoclonal anti-amyloid antibody treatment, in Austria and in Vienna was investigated by reflecting further parameters, such as the share of patients who go through the screening process or qualify for biomarker tests and treatment. The parameters, derived from published meta-analyses, are reasonably applicable to Austria, given that its healthcare infrastructure level is comparable to that of the western European countries reported in the literature [4, 6, 7].

In the memory clinic of the Vienna General Hospital, one of the few clinics in Vienna that provides biomarker-based AD diagnosis, the number of treatment-eligible patients for an upcoming anti-amyloid antibody treatment was investigated from its patient register. First, the number of patients 50–90 years old with MCI was estimated using a mini-mental state examination score of 22 as the screening cut-off according to appropriate use recommendations [8]. In addition, from those who have MCI, the number of patients with biomarker-confirmed brain amyloid positivity was determined using amyloid-PET scan or cerebrospinal fluid (CSF) analysis.

## Results

The estimates of Aβ-positive prodromal AD patients in Austria and Vienna are 193,500 and 34,700 patients, respectively (Table 1). Austria is expected to have a patient demand of about 61,200 patients who are indicated for an upcoming AD treatment, in Vienna the demand is expected to be around 11,100 patients (Table 2).

**Table 1:** Estimates of Aβ-positive prodromal AD in Austria and in Vienna in thousands (uncertainty range)

	Austria			Vienna		
*Age* *(in years)*	Male	Female	Sum	Male	Female	Sum
60–64	8.4 (3.7–17.4)	8.6 (3.8–17.9)	17 (7.6–35.3)	1.5 (0.7–3.1)	1.6 (0.7–3.3)	3.1 (1.4–6.4)
65–69	9 (5.2–15.9)	10 (5.8–17.6)	19 (11–33.5)	1.5 (0.9–2.7)	1.8 (1.1–3.2)	3.4 (1.9–5.9)
70–74	9.9 (6.8–14.4)	11.6 (8.1–17)	21.5 (14.9–31.4)	1.7 (1.2–2.5)	2.2 (1.5–3.2)	3.9 (2.7–5.7)
75–79	12.4 (7.8–19.2)	15.9 (9.9–24.5)	28.3 (17.7–43.7)	2.4 (1.5–3.6)	3.3 (2.1–5.1)	5.7 (3.5–8.8)
80–84	20.6 (12.4–32.1)	28.9 (17.4–45.1)	49.5 (29.9–77.2)	3.7 (2.2–5.8)	5.5 (3.3–8.6)	9.2 (5.6–14.4)
85–89	13.4 (9.2–18.3)	22.7 (15.6–30.9)	36.1 (24.7–49.2)	2 (1.3–2.7)	3.5 (2.4–4.8)	5.5 (3.7–7.5)
90+	6.5 (4.5–8.8)	15.6 (10.8–21.3)	22.1 (15.3–30.1)	1.1 (0.7–1.4)	2.9 (2–3.9)	3.9 (2.7–5.4)
*Sum*	80.2 (49.6–126.1)	113.3 (71.4–174.3)	193.5 (121–300.4)	13.8 (8.5–21.9)	20.8 (13.1–32.2)	34.7 (21.6–54)

**Table 2:** Expected patient demand at each stage of the patient journey in Austria and Vienna for individuals aged 50+ years in thousands*

	Austria			Vienna		
	Male	Female	Sum	Male	Female	Sum
*Age 50+ years*	1777.9	2018.5	3796.4	325.7	386.0	711.8
*Cognitive screening*	1422.3	1614.8	3037.1	260.6	308.8	569.4
*Screening positive for MCI*	151.1	193.0	344.2	27.0	36.2	63.2
*Evaluation by specialist*	75.6	96.5	172.1	13.5	18.1	31.6
*Biomarker testing*	68.0	86.9	154.9	12.1	16.3	28.4
*Positive for biomarkers*	32.4	44.0	76.5	5.7	8.2	13.9
*Treatment indicated*	25.9	35.2	61.2	4.6	6.5	11.1

*MCI *mild cognitive impairment

*The patient demand in each stage of the patient journey was estimated using parameters from published meta-analyses [4, 6, 7]

The analysis of the patient registry at the Vienna General Hospital showed on average 52.8 treatment-eligible patients per year, which is about 10% of the total number of patients visiting the memory clinic every year.

## Discussion

Compared to the prevalence estimates based on published epidemiological data, the actual number of treatment-eligible patients registered in the memory clinic is only a fraction of the expected population.

The complex and laborious process of the diagnosis and the lack of causal treatment of the disease are considered discouraging factors for patients and doctors to perform an early and exact diagnosis, resulting in a huge underdiagnosis of AD in the population [9]. Epidemiological differences between Austria and the literature, especially in education level, may contribute to possible inaccuracies in estimations [6].

## Perspectives

Given the capacity of Austria’s healthcare system, including factors such as hospital beds and magnetic resonance imaging (MRI) scanners, it is anticipated that Austria will face similar limitations in treating AD patients as those encountered by western European countries, such as France and Germany [4, 10]. In attempts to extend the availability of anti-amyloid antibody treatment to a wider population in the upcoming years, several challenges must be addressed:

### Identifying patients:

In order to identify patients who would benefit from the upcoming treatment, it has been suggested to implement MCI screening into routine practice in primary care. Despite the interest of the patients willing to be informed about their cognitive state and the awareness of the primary care provider about the importance of AD screening, the use of dementia screening in the primary care setting remains low [11].

### Biomarker testing:

A confirmation of amyloid-beta plaques through amyloid PET or biomarker analysis from CSF along with a thorough clinical and neuropsychological assessment is essential to identify the target patients for such treatment [8]; however, the current resources of dementia specialists, neuropsychological assessment, amyloid PET scan, and CSF biomarker analysis are insufficient to integrate the treatment into general clinical practice [4]. In the future, blood-based biomarkers, combined with cognitive assessment of the patient, could potentially offer a more resource-efficient alternative [12].

### Infusion delivery:

Patients who qualify for the disease-modifying treatment will receive the medication intravenously every 2 weeks (lecanemab) or every 4 weeks (donanemab) [1, 2]. The current capacity of the healthcare system to deliver the infusion is not sufficient to handle the upcoming demand [4].

### Amyloid-related imaging abnormalities (ARIA) management:

To manage the frequent incidence of ARIA as a side effect of monoclonal anti-amyloid treatment, patients receiving the treatment will need to undergo a routine MRI scan. Current prescription guidelines for lecanemab in the USA advise an MRI scan prior to commencing treatment and subsequently before the 5th, 7th, and 14th infusion. In the occurrence of ARIA, even more frequent MRI monitoring would be required [8].

## Conclusion

The development of the first disease-modifying treatment for AD has opened a new era in AD treatment. Epidemiological analysis based on published meta-analyses suggests a massive number of potential patients who would benefit from the upcoming treatment; however, only a fraction of the expected patient demand is actually registered in the memory clinics. In order to identify target patients and make the treatment available to the general population, the stakeholders must work together to expand the MCI screening in primary care and increase the capacity of the healthcare system for biomarker testing, infusion delivery and ARIA management.
